# Network dysfunction of sadness facial expression processing and morphometry in euthymic bipolar disorder

**DOI:** 10.1007/s00406-023-01649-z

**Published:** 2023-07-27

**Authors:** Alessandro Miola, Nicolò Trevisan, Margherita Salvucci, Matteo Minerva, Silvia Valeggia, Renzo Manara, Fabio Sambataro

**Affiliations:** 1 Department of Neuroscience (DNS), University of Padova, Via Giustiniani 5, Padua, Italy; 2 Padova Neuroscience Center, University of Padova, Padua, Italy

**Keywords:** Bipolar disorder, Emotional processing, Social cognition, Voxel-based morphometry, ROI-based morphometry, Network analysis

## Abstract

Facial emotion recognition (FER), including sadness, is altered in bipolar disorder (BD). However, the relationship between this impairment and the brain structure in BD is relatively unexplored. Furthermore, its association with clinical variables and with the subtypes of BD remains to be clarified. Twenty euthymic patients with BD type I (BD-I), 28 BD type II (BD-II), and 45 healthy controls completed a FER test and a 3D-T1-weighted magnetic resonance imaging. Gray matter volume (GMV) of the cortico-limbic regions implicated in emotional processing was estimated and their relationship with FER performance was investigated using network analysis. Patients with BD-I had worse total and sadness-related FER performance relative to the other groups. Total FER performance was significantly negatively associated with illness duration and positively associated with global functioning in patients with BD-I. Sadness-related FER performance was also significantly negatively associated with the number of previous manic episodes. Network analysis showed a reduced association of the GMV of the frontal–insular–occipital areas in patients with BD-I, with a greater edge strength between sadness-related FER performance and amygdala GMV relative to controls. Our results suggest that FER performance, particularly for facial sadness, may be distinctively impaired in patients with BD-I. The pattern of reduced interrelationship in the frontal–insular–occipital regions and a stronger positive relationship between facial sadness recognition and the amygdala GMV in BD may reflect altered cortical modulation of limbic structures that ultimately predisposes to emotional dysregulation. Future longitudinal studies investigating the effect of mood state on FER performance in BD are warranted.

**Supplementary Information:**

The online version contains supplementary material available at 10.1007/s00406-023-01649-z.

## Introduction

Bipolar disorder (BD) is a severe mental disorder characterized by alterations in emotional regulation [1] representing one of the ten leading causes of disability worldwide [2]. The two most severe subtypes of BD are bipolar disorder type I (BD-I) and bipolar disorder type II (BD-II), which differ in the presence of manic/mixed vs hypomanic episodes [3]. In this context, there is still a debate on ways of subdividing the broad concept of manic-depressive illness introduced by Emil Kraepelin [4–6]. Indeed, BD-II has been proposed to lie within a “BD spectrum” between BD-I and major depressive disorder (MDD) [7, 8], while a single-dimensional BD construct has been proposed [5, 9]. The challenging differentiation between BD subtypes is not just a nosographic issue, but it is relevant to disease management and prognosis [10, 11].

BD-I has more severe symptoms during related mood and a higher level of incapacity during depression compared to BD-II, which presents long-lasting depression [12]. Although previous reports concluded that patients with BD-I and BD-II may differ little in proneness to depressive states [13], recent evidence suggests that patients with BD-II showed higher levels of long-term morbidity characterized by longer and more prominent depressions, a course sequence of depression-[hypo]mania (DM), fewer hospitalizations, and greater risk of suicidal behavior over time compared to those with BD-I [14].

On the neuropsychological level, alterations in social cognition, including facial emotion recognition (FER), which is the ability to identify emotional states from facial expressions [15], can interfere with interpersonal relationships and represents a determinant of the decline of daily functioning [16]. The impairment of emotional perception in BD is moderate and stable [17], and it has been proposed as a trait marker and a possible endophenotype of BD [18]. Indeed, some studies found that patients with BD have a *general* deficit in FER, measured as lower accuracy and/or longer response time [19–22]. Conversely, other studies found *selective* impairments with a deficit in recognizing single emotions [23–27]. In particular, the impairment of sadness processing has been shown to be a critical alteration of emotional recognition in BD. First, a previous study investigating emotion recognition in unaffected relatives of patients with BD found that the offspring of these patients, who are at high risk for the disorder, perform worse than the matched healthy controls (HC) in labeling sad and angry faces, and within individuals at high risk for BD, symptomatic children make more errors than those asymptomatic in labeling sad but not angry or fearful faces [28]. Some investigations explored the association between mood state and FER. Manic patients exhibited an impairment of sadness-related FER that increased with emotional intensity, and this was associated with altered activation of the limbic and the frontal circuitry implicated in facial emotion processing [29, 30]. Moreover, in comparison with depressed patients with BD and controls, manic patients with BD revealed fusiform gyrus hyperactivation in response to sad faces [29]. Conversely, depression in MDD and BD was associated with a negative mood-congruent bias, with a tendency to misinterpret neutral as sad faces and happy as neutral faces, with the severity of depression affecting the overall FER performance [31]. Notably, a study on mood congruency bias in patients with depression (> 70% BD) showed a worse performance in sadness-related FER than controls [32], with patients with BD having a stronger propensity to perceive emotional valence of negative compared to positive facial expressions [33]. During depressive episodes, patients with BD displayed hippocampal hyperactivation during mild facial sadness processing compared to controls and MDD [34]. These selective emotion recognition abnormalities have been hypothesized to contribute to affective symptoms, including persistent sadness, apathy, and anhedonia [32], and to a general “pessimistic outlook” [33]. Interestingly, a previous meta-analysis reported an association of impaired sadness recognition in BD-I, which was marginally significant when including all subtypes of BD [35].

The brain network involved in FER is composed of several regions of the brain with specific functions and entails the amygdala, involved in the arousal of negative emotions [36–38]; the hippocampus, responsible for the recall and regulation of emotional memories [37, 39]; the insula, involved in the modulation of the arousal level [37, 40]; the anterior cingulate cortex, involved in the reward and punishment mechanisms [36, 37, 41]; the fusiform gyrus, responsible for the recognition of the invariant aspect of a face [36, 37, 42]; the prefrontal cortex (PFC), including the superior, medial, and inferior frontal gyrus [36, 42], with a role in the integration of emotion and cognition [43]. A previous large multi-site study investigating morphometric differences in subcortical structures implicated in emotional processing in BD revealed volumetric reductions in patients in the hippocampus and thalamus. However, no differences were found when comparing BD subtypes [44]. The neuroimaging literature has also investigated the morphometry of the brain regions underlying FER impairments in BD. A previous MRI investigation revealed that compared with HC, BD-I performs worse in FER and has reduced gray matter volume (GMV) in the left orbitofrontal cortex, the superior temporal pole, and the insula, and shows a correlation between FER performance and right middle cingulate gyrus GMV [45]. Our group has also demonstrated a loss of GMV in the temporal–occipital regions in BD-I that was correlated with impaired general facial emotional processing [22].

On the other hand, structural covariance, a statistical measure that reflects the relationship between inter-individual structural differences in a property of a specific brain region (e.g., brain volume, cortical thickness, etc.) with inter-individual differences of the same index in a distinct region [46], has been shown to be altered in patients with BD [22, 47, 48]. In particular, we found that patients with BD-I had reduced structural covariance in a prefrontal-temporal-occipital network, and this was associated with increased severity of the disorder, poorer executive functions, and impaired emotional processing [22].

Although growing evidence emphasizes that patients with BD even when euthymic suffer from trait-associated FER impairment [15, 49], the neuroanatomical basis for general and selective FER impairments in euthymic patients with BD, its impact on morbidity and daily functioning, and the differences between the BD subtypes remain to be clarified. The use of univariate analysis, powerful in capturing the variance attributable to a single variable but not when dealing with the complex relationship among multiple measures (regional GMV and behavioral performance) may have contributed to the lack of findings. For this reason, network models have been introduced as alternative approaches for the study of the relationship between variables associated with mental disorders [50–52]. Networks are composed of nodes, representing the observed variables, and edges, indicating their connections. Network centrality indexes can be computed to identify the importance of each node in the network [53]. Network analysis is a data-driven approach, which provides a graphic representation of the complex interrelationships among different types of variables [54].

This approach has been commonly used for studying behavioral variables encompassing psychopathological dimensions [55] and cognitive domains [56, 57]. Interestingly, network analysis methods have recently been used to combine behavioral variables (i.e., symptoms, traits, and cognitive abilities) and their related structural and functional neural correlates in joint networks to characterize their relationship in psychiatric and neurodevelopmental conditions, including depression [58], autism [59], and children and adolescents with learning disabilities [60]. The use of this method has several advantages over traditional approaches. The study in the same analytical paradigm of brain and behavioral data, which often do not correspond to a simple and reductionist one-to-one relationship, allows easier visualization and a simultaneous estimation of the complex pattern of relationship between behavioral and structural properties of the brain [60].

We hypothesized that euthymic BD-I could have impaired emotional processing, particularly for sadness, and that this could be related to a reduced interrelationship between the brain regions implicated in emotional processing and recognition. We also hypothesized that altered FER could be associated with clinical outcomes and functioning. For this reason, in this study, we assessed FER and brain morphometry of the regions implicated in this process and performed a network analysis of these variables in distinct BD subtypes.

## Materials and methods

### Participants

Fifty-one patients with BD and 45 HC were recruited from the psychiatric ward and the outpatient service of the Padua University Hospital. The structured clinical interview for DSM-5-Patient Edition (SCID-5) was used for diagnosis and patients were included if on stable treatment for at least 1 month. A family history of severe mental illness or a current diagnosis of psychiatric disorders or drug treatment (excluding contraceptive pills in women) were exclusion criteria for HC. Participants were excluded if they were younger than 18 or older than 65 years, if they had a lifetime drug dependence, a history of alcohol or drug abuse in the six months before the study, previous traumatic head injury with loss of consciousness, past or present major medical illness, neurological disorders, and mental retardation. Of the initial sample, three patients were excluded: one for a panic attack during the scan and two for vascular lesions on the MRI. A final sample of 48 patients with BD (20 BD-I and 28 BD-II) and 45 HC were enrolled in the study. Written informed consent was obtained from all participants after a complete explanation of the study. The local Ethics Committee authorized this study, and the Helsinki Declaration of 1975 guidelines were followed.

### Clinical assessment

The Montgomery–Asberg Depression Rating Scale (MADRS) [61], the 17‐item Hamilton Rating Scale for Depression (HAM-D) [62], the Hamilton Rating Scale for Anxiety (HAM-A) [63], and the Young Mania Rating Scale (YMRS) [64] were used to evaluate the severity of affective symptoms. Psychotic symptoms were assessed using the Positive and Negative Syndrome Scale (PANSS) [65]. The general psychosocial functioning was evaluated using the Global Assessment of Functioning (GAF) scale [66]. A detailed history of mood disorders was collected, including illness duration, age of onset, familiarity for BD, the number of lifetime affective (depressive, manic, mixed, and hypomanic) episodes, and past occurrence of psychotic symptoms. Data on the current drug treatments, measured using the defined daily dose [67], and serum lithium levels and their duration were also collected.

### FER task

The FER task was administered to evaluate emotional processing [68, 69] using PEBL software (http://pebl.sourceforge.net/). During the FER task, 140 emotion-expressing faces were presented. Four types of expressions were displayed in pseudo-randomized order: sadness (*n* = 40), disgust (*n* = 40), anger (*n* = 40), and neutral (*n* = 20). Participants were asked to identify as quickly as possible the emotion expressed by a face by pressing a button on the labels presented at the bottom of the screen with a touchscreen device. Accuracy and reaction time were recorded. The FER performance calculated as the ratio between the percent accuracy and the mean reaction time [70] for all (FER-total) and individual emotions (FER-sadness, FER-anger, FER-disgust, FER-neutral) was used to estimate the efficiency of emotional processing, which is the speed at which emotions are correctly identified [71]. Given our strong a priori hypotheses on sadness processing, our analysis was limited to the performance during the FER for sad and neutral conditions, the latter being a control condition.

### Image acquisition

High-resolution structural data were acquired using a 3 T MR-scanner (3 Tesla Philips Ingenia) with a 32-channel quadrature head coil. Each participant underwent whole-brain 3D-T1 magnetization-prepared rapid gradient-echo sequence in the sagittal plane with the following parameters: TR/TE = 6676 ms/3 ms, FOV = 240 mm; flip-angle = 8°, resolution = 1.0 × 1.0 × 1.0mm^3^; number of slices = 181. Any abnormalities in the brain were excluded after evaluation by an expert neuroradiologist (RM).

### Voxel-based morphometry (VBM)

Structural MRI data were preprocessed using the Computational Anatomy Toolbox for SPM (CAT12) (http://www.neuro.uni-jena.de/cat/), a toolbox running within the Statistical Parametric Mapping analysis package (SPM12, http://www.fil.ion.ucl.ac.uk/spm/software/spm12/). After the initial data quality check (to avoid critical artifacts such as head motion, ghosting, and stripes that could potentially affect the results), each participant’s T1 image was reoriented to the ACPC and then was spatially normalized and segmented into gray and white matter and cerebrospinal fluid based on the maximum a posteriori estimation. After data preprocessing, modulated normalized gray and white matter volumes were smoothed using an 8-mm Full-Width Half-Maximum (FWHM) Gaussian kernel. We applied a 0.2 absolute masking threshold.

**Fig. 1:**
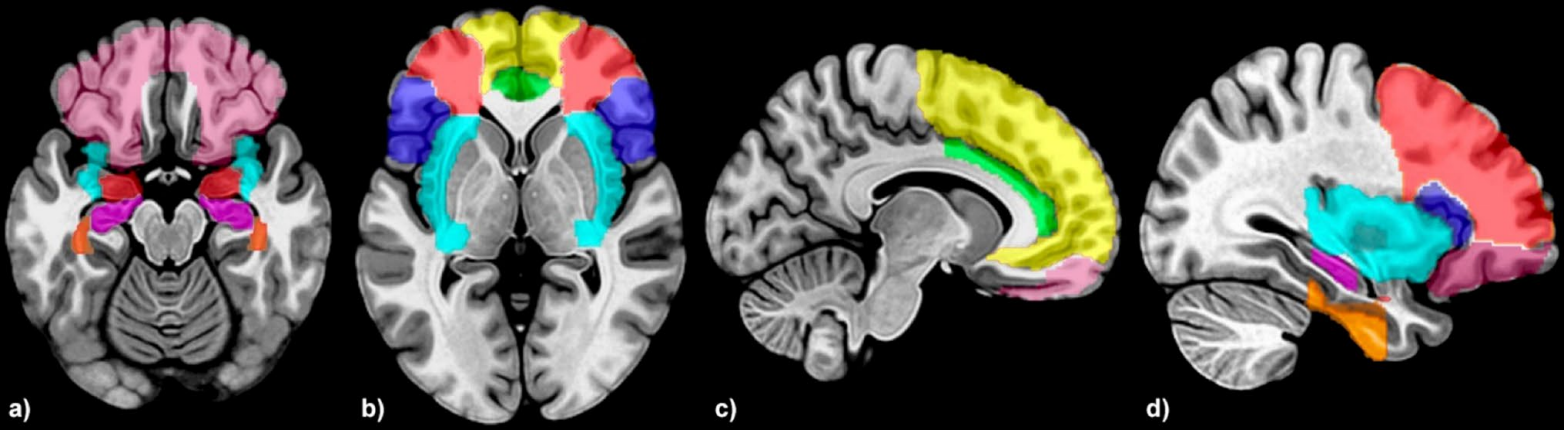
Brain regions involved in facial emotion recognition (FER). Gray matter volume was estimated in regions of interest (ROIs) using voxel-based morphometry and averaged across the hemispheres. ROIs drawn from n30r83 Hammersmith atlas are displayed in axial (**a**, **b**) and sagittal (**c**, **d**) projections of the Montreal Neurological Institute (MNI) template: orbitofrontal cortex (pink), amygdala (dark red), fusiform gyrus (orange), insula (cyan), hippocampus (violet); anterior cingulate cortex (green); superior frontal gyrus (yellow); middle frontal gyrus (light red); inferior frontal gyrus (blue)

All volumetric images underwent quality control for intersubject homogeneity and visual inspection procedure for potential newly introduced artifacts. Given our interest in the structural covariance of GMVs, only these images were included in our analysis. The total intracranial volume (TIV) was estimated to take into account the variability of brain size. Finally, the GMV of each of the nine bilateral regions of interest (ROIs) drawn from the n30r83 Hammersmith atlas (http://brain-development.org/brain-atlases/adult-brain-maximum-probability-map-hammers-mith-atlas-n30r83-in-mni-space/) was estimated and averaged between the two hemispheres. Given our strong a priori hypothesis, we focused on the brain regions involved in the processing of sad emotions and implicated in FER, according to previous literature: the amygdala, hippocampus, insula, anterior cingulate cortex, orbitofrontal cortex, fusiform gyrus, superior frontal gyrus, medial frontal gyrus, and inferior frontal gyrus (see before, Fig. 1).

### Statistical analysis

For the analysis of socio-demographic and clinical data among diagnostic groups, we used χ2-tests for categorical data and one‐way ANOVA for continuous variables, with pairwise χ^2^/Tukey post hoc comparisons in case of statistical significance. The FER-total, as well as the FER-sadness, and the FER-neutral scores among the three diagnoses (BD‐I, BD‐II, and HC) were compared using ANOVA and repeated-measures ANOVA with planned pairwise contrasts (FER-sadness vs. FER-neutral for each diagnosis) using the Bonferroni correction for the number of comparisons (*p* = 0.05/6 = 0.008, 3 between-group comparisons × 2 emotions = 6), respectively. A voxel-wise general linear model (GLM) with TIV and age as covariates was used to compare GMV among the three diagnostic groups (BD-I, BD-II, and controls) using pairwise post hoc t-tests. For each patient group, clinical variables were correlated with FER scores using Pearson’s and Spearman's correlation appropriately. Statistical analysis was performed using JAMOVI (Version 1.2) (https://www.jamovi.org) and R (http://www.rstudio.com/). We used a false discovery rate correction for multiple comparisons for ROI comparisons across diagnoses. The level of significance was set to *p* < 0.05 for all tests.

### Network analysis

The relationship between the FER-sadness and regional GMV was analyzed using network analysis, a relatively novel method for exploring complex patterns of relationships and obtaining a visualization of the network structure of variables. For each sample, a network analysis including 11 nodes was performed: 2 FER scores (FER-sadness and FER-neutral as a control variable) and 9 GMV ROIs. Partial correlations between variables, obtained after partialling out all the other variables, were represented by the ‘edges’ connecting the nodes [72]. We also computed three ‘centrality measures’ for each node [73]: betweenness, which is the number of times that a node is involved in the shortest path between two other nodes, represents the control of the information flow in the network [74, 75]; the closeness, which is the average distance from that node to all other nodes in the network, shows the likelihood for the information to “flow” from a specific node through the entire network both directly and indirectly [75, 76]; strength centrality is calculated as the sum of the edges connected to a node, each one weighted with its own thickness [73, 75]. A Graphical Gaussian Model of the data was fit using the EBICglasso estimator. The stability of the results was verified using a bootstrapping procedure that produced the 95% confidence interval of each edge and the average edge value over 5000 resamplings. Network analyses were carried out using JASP version 0.14.1 (JASP team 2020). We compared network structure and centrality measures between diagnoses using the Network Comparison Test (NCT), which is a two-tailed permutation test on pairwise differences (5000 resamplings). Network structure differences were compared using three invariance measures: network structure invariance, global strength invariance, and edge invariance [77, 78]. The level of significance for all analyses was set to *p* < 0.05.

## Results

### Socio-demographic and clinical data

Age, sex, and handedness did not differ among groups (all *p*’s > 0.1). Patients with BD-I had a significantly higher occurrence of past psychotic symptoms (*p* < 0.001), lower GAF scores (*p* = 0.004), a lower number of past depressive (*p* = 0.033) and hypomanic (*p* = 0.004) episodes compared to those with BD-II. In addition, patients with BD-II had significantly higher use of antidepressants (*p* < 0.001) and lower use of antipsychotics (*p* = 0.007) compared to patients with BD-I. Illness duration, HAM-D, HAM-A, MADRS, and YMRS scores, familiarity for BD, use of anticonvulsants and lithium, and the current plasma lithium levels did not show any significant difference between the patient groups (all *p*’s > 0.1). Lastly, there were no psychiatric comorbidities in the recruited patients with BD-I and BD-II. The socio-demographic and clinical characteristics of the samples are summarized in Table 1.

**Table 1:** Socio-demographic and clinical characteristics of the sample

Characteristics	BD-I (*N* = 20)	BD-II (*N* = 28)	HC (*N* = 45)	*F* or *χ*^2^	*p*
Age (years), mean ± SD	45.5 ± 12.6	38.9 ± 12.6	40.1 ± 12.8	1.745	0.186
Males, *n* (%)	13 (65.0)	19 (67.9)	23 (58.97)	0.589	0.745
Duration of illness (years), mean ± SD	16.6 ± 10.1	12.8 ± 10.7		1.239	0.222
Childhood onset, * n* (%)	3 (15.0)	11 (39.3)		− 1.852	0.070
Previous psychotic symptoms, * n* (%)	11 (55.0)	10 (36.0)		4.899	< 0.001
Familiarity for BD, * n* (%)	15 (75.0)	18 (64.3)		0.778	0.441
Number of past episodes					
Depressive (*N* = 0/1/2+)	6/0/12	1/2/19		6.820	0.033
Manic (*N* = 0/1/2+)	2/8/8	20/0/0		30.707	< 0.001
Hypomanic (*N* = 0/1/2+)	11/3/4	2/6/12		11.156	0.004
Mixed (*N* = 0/1/2+)	14/4/0	20/0/0		5.200	0.023
HAMD, mean ± SD	2.83 ± 5.52	1.69 ± 2.25		0.900	0.374
HAMA, mean ± SD	3.33 ± 6.21	1.45 ± 1.90		1.389	0.173
MADRS, mean ± SD	3.67 ± 7.23	2.17 ± 4.24		0.827	0.413
YMRS, mean ± SD	3.00 ± 6.70	1.09 ± 2.44		1.248	0.220
GAF, mean ± SD	65.00 ± 23.37	80.58 ± 8.41		− 3.076	0.004
Current pharmacotherapy					
Antidepressants, *n* (%)	6 (30.0)	22 (78.6)		− 3.365	< 0.001
Antipsychotics, *n* (%)	15 (75.0)	10 (35.7)		2.686	0.007
Anticonvulsants, *n* (%)	6 (30.0)	4 (14.3)		1.322	0.187
Lithium, *n* (%)	19 (95.0)	28 (100)		− 1.196	0.230
Lithium treatment duration (months), mean ± SD	89.5 ± 118.9	28.3 ± 40.6		2.531	0.015
Lithium plasma level (mmol/L), mean ± SD	0.550 ± 0.270	0.522 ± 0.170		0.427	0.672

An ANOVA and a chi-square test were performed to compare age and sex among groups. Two sample *t*-tests and chi-square tests were performed for continuous and categorical variables, respectively, when only two groups were compared

*HAMD* Hamilton Rating Scale for Depression, *HAMA* Hamilton Rating Scale for Anxiety, *MADRS* Montgomery–Asberg Depression Rating Scale, *YMRS* Young Mania Rating Scale, *GAF* Global Assessment of Functioning, *SD* standard deviation, *BD-I* bipolar disorder type I, *BD-II* bipolar disorder type II, *HC* healthy controls

### ROI-based VBM analysis

The average GMV of each ROI did not show significant differences among groups (see supplementary materials, Table S.1).

### FER task

**Fig. 2:**
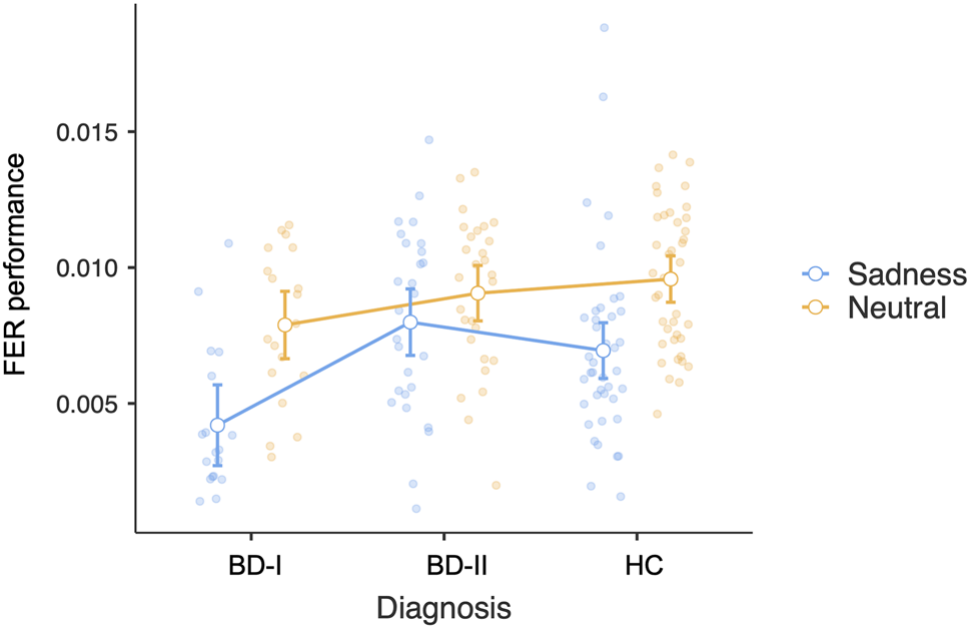
The performance of facial emotion recognition of sadness (FER-sadness) is altered in BD-I relative to BD-II and HC. BD-I and HC show reduced FER during the presentation of sadness compared to neutral, in contrast with BD-II, who have similar performance independent of facial emotion. The colored dots indicate FER performance for each diagnostic group and emotion (sadness in yellow, and neutral in blue); the white dots indicate the mean, and the bars the 95% confidence intervals for each emotion and diagnosis. FER performance scores are calculated as the ratio between the % accuracy and the mean reaction time. BD-I, bipolar disorder type I; BD-II, bipolar disorder type II; HC, healthy controls

**Fig. 3:**
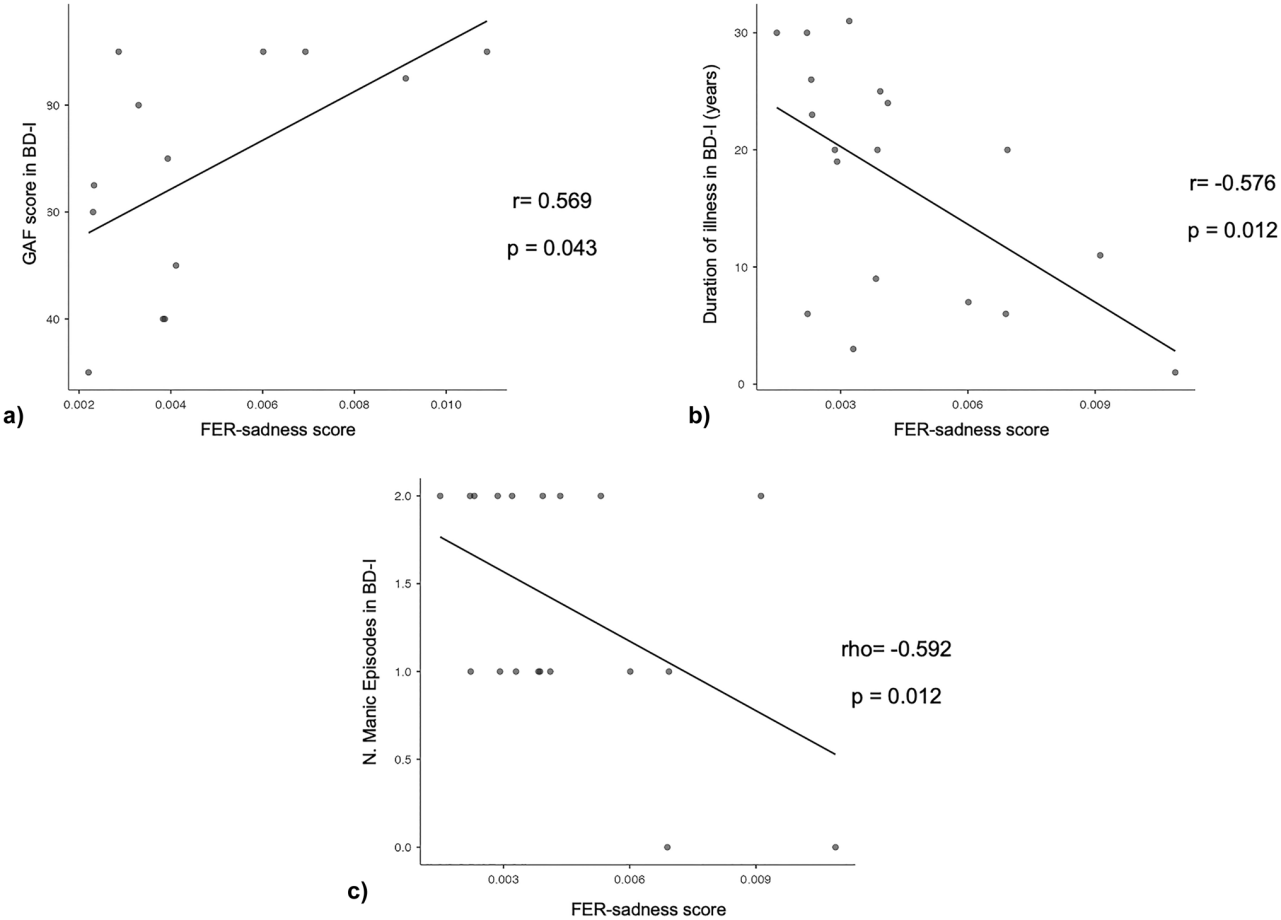
Facial emotion recognition of sadness (FER-sadness) correlates with clinical characteristics and functioning in patients with bipolar disorder type I (BD-I). Scatterplots represent the relationship between FER-sadness scores (% accuracy/mean reaction time) and the Global Assessment of Functioning (GAF) scale scores (**a**), duration of illness (**b**) and the number of previous manic episodes (**c**) with FER-sadness performance in patients with bipolar disorder type I (BD-I). Pearson’s *r* or Spearman’s rho and *p* values for each correlation are reported on the right-hand side of each scatter plot

There was an effect of diagnosis on FER-total scores [*F*(2, 90) = 8.928, *p* < 0.001], with patients with BD-I having significantly worse performance compared to patients with BD-II and HC (*p* < 0.001), and there were no differences between patients with BD-II and HC (*p* = 0.9). In patients with BD-I, the FER-total scores were significantly correlated with the duration of the illness (*r* = − 0.543, *p* = 0.02) and the GAF scores (*r* = 0.656, *p* = 0.015). The emotion-by-diagnosis ANOVA confirmed the effect of diagnosis (*p* < 0.001) with the poorest performance in patients with BD-I relative to those with BD-II and HC (all *p*’s < 0.001), and showed the effect of emotion [*F*(1, 84) = 31.02, *p* < 0.001] with the poorest performance for sadness relative to neutral [*t*(84) = 5.57, *p* < 0.001], and a marginal significance for their interaction [*F*(2, 84) = 2.68, *p* = 0.07] with planned comparisons showing Bonferroni-corrected significance for FER-sadness vs FER-neutral difference comparing patients with BD-I [*F*(2, 84) = 4.081, *p* < 0.001] with HC [*F*(2, 84) = 4.226, *p* < 0.001] but not with patients with BD-II [*F*(2, 84) = 1.433, *p* = 0.156] (see Fig. 2). FER-sadness was significantly correlated with the duration of illness (*r* = − 0.576, *p* = 0.012), the GAF score (*r* = 0.569, *p* = 0.043), as well as with the number of previous manic episodes (rho = − 0.592, *p* = 0.012) in patients with BD-I (see Fig. 3). In patients with BD-I, FER-total, and FER for each emotion scores did not correlate with the antidepressant dose or with plasma lithium levels (all *p*’s > 0.05). Conversely, in patients with BD-I, FER-total, and FER-sadness scores were significantly correlated with the dose of antipsychotics (rho = − 0.561, *p* = 0.02, and rho = − 0.508, *p* = 0.04, respectively). We did not find any significant correlation between FER scores and clinical variables in patients with BD-II.

### Network analysis

**Fig. 4:**
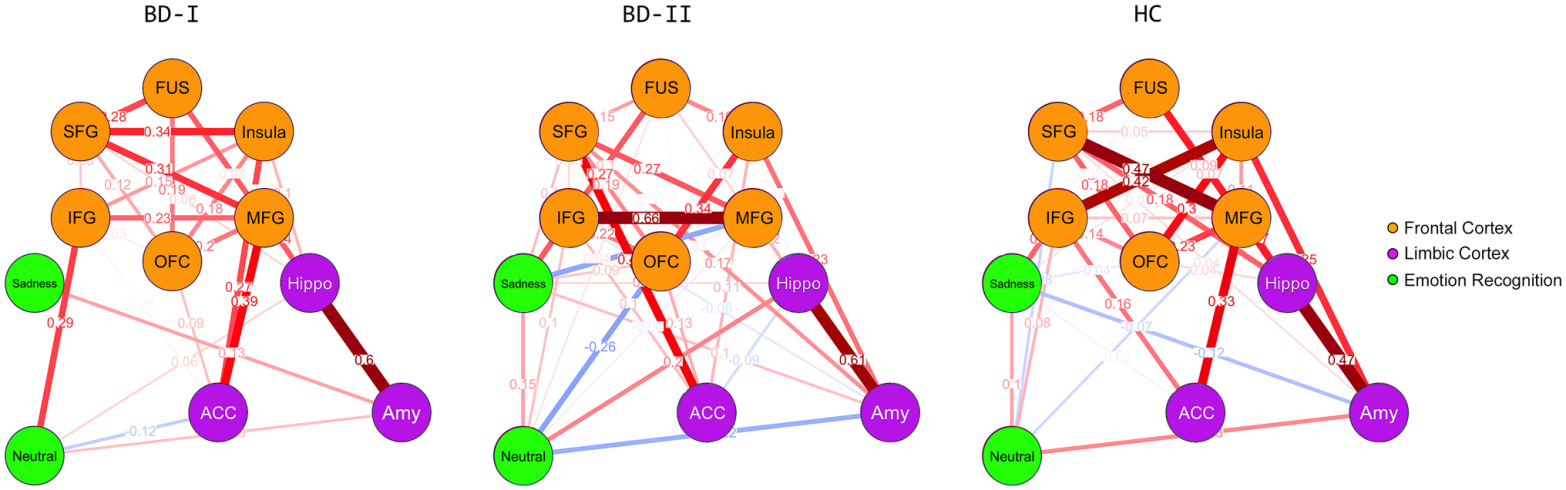
Network plot of the relationship between facial emotion recognition and brain morphometry of the regions implicated in facial emotion recognition (FER) for each diagnostic group. The thickness of the edge represents the strength of the correlation, and its color represents the sign of the correlation. The red lines indicate positive associations; blue lines indicate negative associations. The nodes are grouped by color: green for emotion recognition: *Sadness* FER-sadness score, *Neutral* FER-neutral score; purple for limbic cortex morphometry: Insula, *Amy* amygdala, *Hippo* hippocampus, *ACC* anterior cingulate cortex; orange for fronto-insulo-temporal cortex morphometry: *FUS* fusiform gyrus, *SFG* superior frontal gyrus, *MFG* medial frontal gyrus, *IFG* inferior frontal gyrus. The FER performance was calculated as the ratio between the % accuracy and the reaction time. Brain morphometry was measured as the average gray matter volume extracted from bilateral regions of interest using voxel-based morphometry. *BD-I* bipolar disorder type I, *BD-II* bipolar disorder type II, *HC* healthy controls

The network for patients with BD-I showed a reduced interrelationship in the frontal–insular–occipital regions (superior frontal gyrus, middle frontal gyrus, inferior frontal gyrus, orbitofrontal cortex, insula, fusiform gyrus) relative to those with BD-II and HC, as well as between FER-sadness and FER-neutral (see Fig. 4). In patients with BD-I, FER-sadness, and FER-neutral were not associated with frontal–insular–occipital regions, and the strength of the FER-sadness–amygdala edge was greater compared to the other groups (HC had a negative sign in this edge). NCT confirmed a significant difference in the network structure invariance between patients with BD-I and HC (*p* < 0.001) as well as between patients with BD-II and HC (*p* < 0.001). In contrast, no differences were observed between the BD subtypes (*p* = 0.85). We did not find any difference in terms of global strength invariance among groups (all *p*’s > 0.1). Finally, the edge invariance test, which compares the edge (connection) strength [77], confirmed a stronger positive relationship between FER-sadness and amygdala GMV in patients with BD-I relative to HC (*p* = 0.005) but not between HC and those with BD-II or between BD subtypes (all *p*’s > 0.1). We did not find any significant differences in centrality measures between diagnoses (see Table S.2 for descriptive statistics on these measures).

## Discussion

In this study, three main findings emerged. First, euthymic patients with BD-I had the poorest performance in recognizing facial emotion expressions, particularly sadness. Second, in those with BD-I, FER performance, specifically for sadness, was correlated with illness duration and GAF scores. Additionally, FER for sadness in patients with BD-I was negatively associated with the number of previous manic episodes. Third, the overall structure of the network of patients with BD-I and patients with BD-II was altered, with a reduced GMV interrelationship in the frontal–insular–occipital regions in those with BD-I. Furthermore, the edge strength between sadness-related FER performance and amygdala GMV was stronger in patients with BD-I compared to the other groups, according to the edge invariance test. Lastly, FER performance during the presentation of sadness was poorer compared to neutral in patients with BD-I and HC but not in those with BD-II.

Although some small studies did not show FER impairments in BD [79, 80], a general impairment of this process is consistent with the previous literature [15, 18, 20–22, 49, 81, 82] and appears to be independent of visuoperceptual problems [19]. Notably, we found that BD-I had the poorest performance for FER, specifically for sadness. A bias towards negative-valanced emotion has been described not only during a mood phase but also in euthymia in patients with BD-I, thus suggesting a trait alteration [83, 84]. Also, facial sadness was rated more intensely by euthymic or mildly depressed patients with BD, with a specific impairment of the microexpression recognition of this emotion [24]. Notably, clinically stable patients with BD had a specific impairment of FER for sadness even when compared to patients with MDD [85]. Cognitive studies investigating negative cognitions in depression have shown that euthymic patients with BD had a negative schema content which is lower relative to patients with depression (BD and MDD) when tested explicitly and increases when tested implicitly [86]. Indeed, euthymic patients with BD may unconsciously compensate for underlying depressogenic cognitions by masking responses to explicit measures but fail to do so with implicit unconscious measures, thus suggesting depression-avoidance defense mechanisms [86].

Only a few studies investigated emotion processing differences between BD subtypes [87]. A small study showed that euthymic patients with BD-II had greater fear recognition compared to manic and euthymic patients with BD-I [88]. However, other studies did not report differences in FER performance between the BD subtypes [26, 89, 90]. These contradictory findings could be related to methodological differences (e.g., facial expression dynamics, performance assessment, facial stimuli standardization, etc.) [49]. Notably, patients with BD-II displayed a similar performance for sadness and neutral FER in contrast with the other groups. This finding is consistent with better psychosocial functioning in patients with BD-II compared with those with BD-I [91], as emerged in our study. The higher number of previous depressive episodes in patients with BD-II relative to those with BD-I (*p* = 0.033), together with the greater amount of depressed/[hypo]manic time spent and the more frequent depression-predominant polarity in patients with BD-II [14], may result in the greater familiarity in recognizing sadness in patients with BD-II. However, these findings need further replication.

Of clinical relevance, the current study revealed that FER and FER-sadness performance were poorer in patients with BD-I with a longer duration of illness and lower GAF scores. FER-sadness was also reduced in patients with BD-I with a higher number of previous manic episodes. In a previous study, the authors found an emotion recognition deficit in low-functioning remitted patients with BD [92]. However, findings are mixed, with some studies unable to identify an association between FER and clinical variables [26, 93]. In contrast, a recent report found that patients with BD-II but not BD-I had difficulties in general FER compared to HC, with an association between poorer performance and shorter disease duration, thus speculating that FER impairment could be an early characteristic of patients with BD-II [89]. Although previous studies explored the possibility that antidepressant medications can affect FER [94, 95], this association remains unclear in BD. Our analysis demonstrated that FER ability appears to be independent of the use of antidepressants as well as of lithium treatment. However, FER performance was negatively correlated with antipsychotic dose. The results of the network analysis provided insight into the relationship between the recognition of sad faces and GMV changes in the brain regions involved in emotion processing in BD subtypes. Our study found a reduced interrelationship between frontal–insular–occipital GMV ROIs in patients with BD-I. These findings are partially in line with a previous study of structural covariance with a multivariate approach that revealed two distinct structural networks: a shared psychotic core, equally reduced in both patients with BD and schizophrenia (SZ) compared to HC, including portions of the medial parietal and temporal–occipital areas, and parts of the middle frontal gyrus and cerebellum, as well as an affective core, more compromised in patients with BD versus those with SZ, that included portions of the temporal and occipital lobes, cerebellum, and frontal gyrus [48].

Furthermore, we found a stronger positive relationship between FER-sadness and amygdala GMV in patients with BD-I relative to HC (*p* = 0.005), as emerged using the edge invariance test. Notably, FER-sadness in patients with BD-I was not associated with GMV in the fronto-insular areas (see Fig. 4). The ability to recognize sadness in patients with BD-I appears to be more dependent on amygdala morphometry rather than on frontal–insular–occipital areas, thus confirming that amygdala alterations may be a crucial feature of the disorder [96, 97]. In BD-I, an altered cortico-limbic circuit could underlie altered emotional processing [98, 99]. In keeping with this, previous functional neuroimaging studies reported a reduction of the connectivity between the amygdala and the ventral-PFC [100], the dorsolateral-PFC [101], and the perigenual anterior cingulate cortex [102, 103]. Overall, our findings support a consensus model in which BD-I results from abnormalities in the structure and function of key emotional control networks leading to decreased connectivity between the ventral-PFC and limbic brain regions, especially the amygdala [104].

This study has some limitations. First, this is a cross-sectional study and causality and developmental inference cannot be made. Second, the patients were taking medications that might have slowed emotional recognition performance and confounded morphometric measures [105]. For ethical and clinical reasons, it would not be realistic to enroll drug-free patients, to completely rule out the effects of the medication on neuropsychological and morphometric analysis.

In conclusion, the current study with an integrated approach using clinical, behavioral, and morphometric data showed that patients with BD-I have poorer performance in facial emotion recognition, specifically for sadness, and that this deficit is associated with impaired daily functioning and mood instability expressed by previous manic episodes. In addition, the network analysis provided evidence to support a model of fronto-limbic dysfunction in sadness processing in patients with BD-I relative to BD-II. Future longitudinal studies are needed to investigate the effect of mood state and psychotropic treatments on FER performance and to address causal inferences between emotional processing, daily functioning, and morbidity.

### Supplementary Information

Below is the link to the electronic supplementary material.

Supplementary file1 (DOCX 24 KB)

## Data Availability

The data that support the findings of this study are available from the corresponding author upon reasonable request.

## References

[1] Miola A, Cattarinussi G, Antiga G (2022). Difficulties in emotion regulation in bipolar disorder: a systematic review and meta-analysis. J Affect Disord.

[2] World Health Organization (2001). The World health report: 2001: mental health: new understanding, new hope.

[3] (2013) Diagnostic and statistical manual of mental disorders: DSM-5^TM^, 5th ed. American Psychiatric Publishing, Inc., Arlington

[4] Nierenberg AA (2019). Bipolar II disorder is not a myth. Can J Psychiatry Rev Can Psychiatr.

[5] Malhi GS, Outhred T, Irwin L (2019). Bipolar II disorder is a myth. Can J Psychiatry.

[6] Parker G (2021). Polarised views about bipolar disorder(s): a critique of the 2020 College guidelines for mood disorders. Aust N Z J Psychiatry.

[7] Akiskal HS (2002). The bipolar spectrum—the shaping of a new paradigm in psychiatry. Curr Psychiatry Rep.

[8] Miola A, Tondo L, Pinna M (2023). Comparison of bipolar disorder type II and major depressive disorder. J Affect Disord.

[9] Malhi GS, Bell E, Boyce P (2020). The 2020 Royal Australian and New Zealand College of psychiatrists clinical practice guidelines for mood disorders: Bipolar disorder summary. Bipolar Disord.

[10] Bega S, Schaffer A, Goldstein B, Levitt A (2012). Differentiating between bipolar disorder types I and II: results from the National Epidemiologic Survey on Alcohol and Related Conditions (NESARC). J Affect Disord.

[11] Tondo L, Miola A, Pinna M (2022). Two bipolar disorders or one? In reply to commentary by Malhi and Bell. Int J Bipolar Disord.

[12] Guzman-Parra J, Streit F, Forstner AJ (2021). Clinical and genetic differences between bipolar disorder type 1 and 2 in multiplex families. Transl Psychiatry.

[13] Pallaskorpi S, Suominen K, Ketokivi M (2015). Five-year outcome of bipolar I and II disorders: findings of the Jorvi Bipolar Study. Bipolar Disord.

[14] Tondo L, Miola A, Pinna M (2022). Differences between bipolar disorder types 1 and 2 support the DSM two-syndrome concept. Int J Bipolar Disord.

[15] Rocca CCDA, van den Heuvel E, Caetano SC, Lafer B (2009). Facial emotion recognition in bipolar disorder: a critical review. Rev Bras Psiquiatr Sao Paulo Braz.

[16] Vlad M, Raucher-Chéné D, Henry A, Kaladjian A (2018). Functional outcome and social cognition in bipolar disorder: is there a connection?. Eur Psychiatry J Assoc Eur Psychiatr.

[17] Kohler CG, Hoffman LJ, Eastman LB (2011). Facial emotion perception in depression and bipolar disorder: a quantitative review. Psychiatry Res.

[18] Derntl B, Habel U (2011). Deficits in social cognition: a marker for psychiatric disorders?. Eur Arch Psychiatry Clin Neurosci.

[19] Bozikas VP, Tonia T, Fokas K (2006). Impaired emotion processing in remitted patients with bipolar disorder. J Affect Disord.

[20] David DP, Soeiro-de-Souza MG, Moreno RA, Bio DS (2014). Facial emotion recognition and its correlation with executive functions in bipolar I patients and healthy controls. J Affect Disord.

[21] Hoertnagl CM, Muehlbacher M, Biedermann F (2011). Facial emotion recognition and its relationship to subjective and functional outcomes in remitted patients with bipolar I disorder. Bipolar Disord.

[22] Miola A, Trevisan N, Merola A (2022). Gray matter volume covariance networks are associated with altered emotional processing in bipolar disorder: a source-based morphometry study. Brain Imaging Behav.

[23] Altamura M, Padalino FA, Stella E (2016). Facial emotion recognition in bipolar disorder and healthy aging. J Nerv Ment Dis.

[24] Branco LD, Cotrena C, Ponsoni A (2018). Identification and perceived intensity of facial expressions of emotion in bipolar disorder and major depression. Arch Clin Neuropsychol Off J Natl Acad Neuropsychol.

[25] de Brito Ferreira Fernandes F, Gigante AD, Berutti M (2016). Facial emotion recognition in euthymic patients with bipolar disorder and their unaffected first-degree relatives. Compr Psychiatry.

[26] Martino DJ, Strejilevich SA, Fassi G (2011). Theory of mind and facial emotion recognition in euthymic bipolar I and bipolar II disorders. Psychiatry Res.

[27] Thaler NS, Strauss GP, Sutton GP (2013). Emotion perception abnormalities across sensory modalities in bipolar disorder with psychotic features and schizophrenia. Schizophr Res.

[28] Hanford LC, Sassi RB, Hall GB (2016). Accuracy of emotion labeling in children of parents diagnosed with bipolar disorder. J Affect Disord.

[29] Chen C-H, Lennox B, Jacob R (2006). Explicit and implicit facial affect recognition in manic and depressed States of bipolar disorder: a functional magnetic resonance imaging study. Biol Psychiatry.

[30] Lennox BR, Jacob R, Calder AJ (2004). Behavioural and neurocognitive responses to sad facial affect are attenuated in patients with mania. Psychol Med.

[31] Gur RC, Erwin RJ, Gur RE (1992). Facial emotion discrimination: II. Behavioral findings in depression Psychiatry Res.

[32] Rubinow DR, Post RM (1992). Impaired recognition of affect in facial expression in depressed patients. Biol Psychiatry.

[33] Lior R, Nachson I (1999). Impairments in judgment of chimeric faces by schizophrenic and affective patients. Int J Neurosci.

[34] Lawrence NS, Williams AM, Surguladze S (2004). Subcortical and ventral prefrontal cortical neural responses to facial expressions distinguish patients with bipolar disorder and major depression. Biol Psychiatry.

[35] Samamé C, Martino DJ, Strejilevich SA (2015). An individual task meta-analysis of social cognition in euthymic bipolar disorders. J Affect Disord.

[36] Gur RC, Schroeder L, Turner T (2002). Brain activation during facial emotion processing. Neuroimage.

[37] Haxby JV, Hoffman EA, Gobbini MI (2002). Human neural systems for face recognition and social communication. Biol Psychiatry.

[38] Purves D, Augustine GJ, Fitzpatrick D et al (2001) Emotions. In: Neuroscience, 2nd edn. Sinauer Associates, MA, USA

[39] Femenía T, Gómez-Galán M, Lindskog M, Magara S (2012). Dysfunctional hippocampal activity affects emotion and cognition in mood disorders. Brain Res.

[40] Craig ADB (2009). How do you feel–now? The anterior insula and human awareness. Nat Rev Neurosci.

[41] Rolls ET (2019). The cingulate cortex and limbic systems for emotion, action, and memory. Brain Struct Funct.

[42] Kesler-West ML, Andersen AH, Smith CD (2001). Neural substrates of facial emotion processing using fMRI. Brain Res Cogn Brain Res.

[43] Beer JS, Knight RT, D’Esposito M (2006). Controlling the integration of emotion and cognition: the role of frontal cortex in distinguishing helpful from hurtful emotional information. Psychol Sci.

[44] Hibar DP, for the ENIGMA Bipolar Disorder Working Group, the Costa Rica/Colombia Consortium for Genetic Investigation of Bipolar Endophenotypes (2016). Subcortical volumetric abnormalities in bipolar disorder. Mol Psychiatry.

[45] Neves MDCL, Albuquerque MR, Malloy-Diniz L (2015). A voxel-based morphometry study of gray matter correlates of facial emotion recognition in bipolar disorder. Psychiatry Res.

[46] Xu L, Groth KM, Pearlson G (2009). Source-based morphometry: the use of independent component analysis to identify gray matter differences with application to schizophrenia. Hum Brain Mapp.

[47] Lapomarda G, Grecucci A, Messina I (2021). Common and different gray and white matter alterations in bipolar and borderline personality disorder: a source-based morphometry study. Brain Res.

[48] Sorella S, Lapomarda G, Messina I (2019). Testing the expanded continuum hypothesis of schizophrenia and bipolar disorder. Neural and psychological evidence for shared and distinct mechanisms. NeuroImage Clin.

[49] Miskowiak KW, Seeberg I, Kjaerstad HL (2019). Affective cognition in bipolar disorder: a systematic review by the ISBD targeting cognition task force. Bipolar Disord.

[50] Borsboom D (2017). A network theory of mental disorders. World Psychiatry.

[51] Borsboom D, Cramer AOJ, Kalis A (2019). Brain disorders? Not really: why network structures block reductionism in psychopathology research. Behav Brain Sci.

[52] McNally RJ (2016). Can network analysis transform psychopathology?. Behav Res Ther.

[53] Monteleone AM, Cascino G (2021). A systematic review of network analysis studies in eating disorders: is time to broaden the core psychopathology to non specific symptoms. Eur Eat Disord Rev.

[54] Galderisi S, Rucci P, Kirkpatrick B (2018). Interplay among psychopathologic variables, personal resources, context-related factors, and real-life functioning in individuals with schizophrenia: a network analysis. JAMA Psychiat.

[55] Robinaugh DJ, Hoekstra RHA, Toner ER, Borsboom D (2020). The network approach to psychopathology: a review of the literature 2008–2018 and an agenda for future research. Psychol Med.

[56] Galimberti C, Bosi MF, Caricasole V (2020). Using network analysis to explore cognitive domains in patients with unipolar versus bipolar depression: a prospective naturalistic study. CNS Spectr.

[57] Karyakina M, Shmukler A (2021). Network analysis of cognitive deficit in patients with schizophrenia spectrum disorders. Schizophr Res Cogn.

[58] Hilland E, Landrø NI, Kraft B (2020). Exploring the links between specific depression symptoms and brain structure: a network study. Psychiatry Clin Neurosci.

[59] Bathelt J, Geurts HM, Borsboom D (2022). More than the sum of its parts: merging network psychometrics and network neuroscience with application in autism. Netw Neurosci Camb Mass.

[60] Simpson-Kent IL, Fried EI, Akarca D (2021). Bridging brain and cognition: a multilayer network analysis of brain structural covariance and general intelligence in a developmental sample of struggling learners. J Intell.

[61] Montgomery SA, Asberg M (1979). A new depression scale designed to be sensitive to change. Br J Psychiatry J Ment Sci.

[62] Hamilton M (1960). A rating scale for depression. J Neurol Neurosurg Psychiatry.

[63] Hamilton M (1959). The assessment of anxiety states by rating. Br J Med Psychol.

[64] Young RC, Biggs JT, Ziegler VE, Meyer DA (1978). A rating scale for mania: reliability, validity and sensitivity. Br J Psychiatry J Ment Sci.

[65] Kay SR, Fiszbein A, Opler LA (1987). The positive and negative syndrome scale (PANSS) for schizophrenia. Schizophr Bull.

[66] Association AP (2000). Diagnostic and statistical manual of mental disorders, 4th edition, text revision.

[67] Nosè M, Barbui C (2008). A simple approach to manage dosages in drug-epidemiology research. Epidemiol Psichiatr Soc.

[68] De Panfilis C, Antonucci C, Meehan KB (2019). Facial emotion recognition and social-cognitive correlates of narcissistic features. J Personal Disord.

[69] Meehan KB, De Panfilis C, Cain NM (2017). Facial emotion recognition and borderline personality pathology. Psychiatry Res.

[70] Vandierendonck A (2017). A comparison of methods to combine speed and accuracy measures of performance: a rejoinder on the binning procedure. Behav Res Methods.

[71] Leathem LD, Currin DL, Montoya AK, Karlsgodt KH (2021). Socioemotional mechanisms of loneliness in subclinical psychosis. Schizophr Res.

[72] Epskamp S, Fried EI (2018). A tutorial on regularized partial correlation networks. Psychol Methods.

[73] Opsahl T, Agneessens F, Skvoretz J (2010). Node centrality in weighted networks: generalizing degree and shortest paths. Soc Netw.

[74] Brandes U (2001). A faster algorithm for betweenness centrality*. J Math Sociol.

[75] Costantini G, Epskamp S, Borsboom D (2015). State of the aRt personality research: a tutorial on network analysis of personality data in R. J Res Personal.

[76] Freeman LC (1978). Centrality in social networks conceptual clarification. Soc Netw.

[77] van Borkulo CD, van Bork R, Boschloo L (2022). Comparing network structures on three aspects: a permutation test. Psychol Methods.

[78] van Borkulo C, Boschloo L, Borsboom D (2015). Association of symptom network structure with the course of [corrected] depression. JAMA Psychiat.

[79] Goghari VM, Sponheim SR (2013). More pronounced deficits in facial emotion recognition for schizophrenia than bipolar disorder. Compr Psychiatry.

[80] Venn HR, Gray JM, Montagne B (2004). Perception of facial expressions of emotion in bipolar disorder. Bipolar Disord.

[81] Ryan KA, Vederman AC, Kamali M (2013). Emotion perception and executive functioning predict work status in euthymic bipolar disorder. Psychiatry Res.

[82] Soeiro-de-Souza MG, Otaduy MCG, Dias CZ (2012). The impact of the CACNA1C risk allele on limbic structures and facial emotions recognition in bipolar disorder subjects and healthy controls. J Affect Disord.

[83] Gopin CB, Burdick KE, Derosse P (2011). Emotional modulation of response inhibition in stable patients with bipolar I disorder: a comparison with healthy and schizophrenia subjects. Bipolar Disord.

[84] Sollier-Guillery M, Fortier A, Dondaine T (2021). Emotions and cognitive control: A comparison of bipolar disorder and schizophrenia. J Affect Disord Rep.

[85] Vederman AC, Weisenbach SL, Rapport LJ (2012). Modality-specific alterations in the perception of emotional stimuli in bipolar disorder compared to healthy controls and major depressive disorder. Cortex J Devoted Study Nerv Syst Behav.

[86] Granger S, Pavlis A, Collett J, Hallam KT (2021). Revisiting the “manic defence hypothesis”: assessing explicit and implicit cognitive biases in euthymic bipolar disorder. Clin Psychol.

[87] Bora E (2018). Neurocognitive features in clinical subgroups of bipolar disorder: a meta-analysis. J Affect Disord.

[88] Lembke A, Ketter TA (2002). Impaired recognition of facial emotion in mania. Am J Psychiatry.

[89] Jensen MB, Kjærstad HL, Coello K (2021). Affective and non-affective cognition in patients with bipolar disorder type I and type II in full or partial remission: associations with familial risk. J Affect Disord.

[90] Summers M, Papadopoulou K, Bruno S (2006). Bipolar I and bipolar II disorder: cognition and emotion processing. Psychol Med.

[91] Dell’Osso B, Dobrea C, Cremaschi L (2017). Italian bipolar II vs I patients have better individual functioning, in spite of overall similar illness severity. CNS Spectr.

[92] Lahera G, Ruiz-Murugarren S, Iglesias P (2012). Social cognition and global functioning in bipolar disorder. J Nerv Ment Dis.

[93] Işık Ulusoy S, Gülseren ŞA, Özkan N, Bilen C (2020). Facial emotion recognition deficits in patients with bipolar disorder and their healthy parents. Gen Hosp Psychiatry.

[94] Harmer CJ, Bhagwagar Z, Perrett DI (2003). Acute SSRI administration affects the processing of social cues in healthy volunteers. Neuropsychopharmacol Off Publ Am Coll Neuropsychopharmacol.

[95] Tranter R, Bell D, Gutting P (2009). The effect of serotonergic and noradrenergic antidepressants on face emotion processing in depressed patients. J Affect Disord.

[96] Blumberg HP, Fredericks C, Wang F (2005). Preliminary evidence for persistent abnormalities in amygdala volumes in adolescents and young adults with bipolar disorder. Bipolar Disord.

[97] Kalmar JH, Wang F, Chepenik LG (2009). Relation between amygdala structure and function in adolescents with bipolar disorder. J Am Acad Child Adolesc Psychiatry.

[98] Bi B, Che D, Bai Y (2022). Neural network of bipolar disorder: toward integration of neuroimaging and neurocircuit-based treatment strategies. Transl Psychiatry.

[99] Bigot M, Alonso M, Houenou J (2020). An emotional-response model of bipolar disorders integrating recent findings on amygdala circuits. Neurosci Biobehav Rev.

[100] Liu H, Tang Y, Womer F (2014). Differentiating patterns of amygdala-frontal functional connectivity in schizophrenia and bipolar disorder. Schizophr Bull.

[101] Radaelli D, Sferrazza Papa G, Vai B (2015). Fronto-limbic disconnection in bipolar disorder. Eur Psychiatry J Assoc Eur Psychiatry.

[102] Furlong LS, Rossell SL, Caruana GF (2021). The activity and connectivity of the facial emotion processing neural circuitry in bipolar disorder: a systematic review. J Affect Disord.

[103] Wang F, Kalmar JH, He Y (2009). Functional and structural connectivity between the perigenual anterior cingulate and amygdala in bipolar disorder. Biol Psychiatry.

[104] Strakowski SM, Adler CM, Almeida J (2012). The functional neuroanatomy of bipolar disorder: a consensus model. Bipolar Disord.

[105] Young W (2009). Review of lithium effects on brain and blood. Cell Transplant.

