# Supplementary material for: Network dysfunction of sadness facial expression processing and morphometry in euthymic bipolar disorder
Source: Eur Arch Psychiatry Clin Neurosci. 2023 Jul 27;274(3):525–36. doi: 10.1007/s00406-023-01649-z (PMC10995000; doi:10.1007/s00406-023-01649-z)
Supplement: Supplementary file 1 — Supplementary file1 (DOCX 24 KB) [file 406_2023_1649_MOESM1_ESM.docx]

**Network dysfunction of sadness facial expression processing and morphometry in euthymic bipolar disorder**

Alessandro Miola^1^, Nicolò Trevisan^1^, Margherita Salvucci^1^, Matteo Minerva^1^, Silvia Valeggia^1^, Renzo Manara^1,2^, Fabio Sambataro^1,2^

^1^ Department of Neuroscience (DNS), University of Padova, Padua, Italy

^2^ Padova Neuroscience Center, University of Padova, Padua, Italy

|  |  | |  | | | |  | | |  |
| --- | --- | --- | --- | --- | --- | --- | --- | --- | --- | --- |
| **Region of interest** | | **Atlas labels** | | **BD-I** | **BD-II** | **HC** | | **F** | **p** | |
| Hippocampus | | Hip | | 2.102 ± 0.115 | 2.129 ± 0.153 | 2.128 ± 0.205 | | 0.057 | 0.944 | |
| Amygdala | | Amy | | 1.755 ± 0.132 | 1.824 ± 0.142 | 1.797 ± 0.175 | | 0.194 | 0.823 | |
| Fusiform Gyrus | | FusGy | | 3.049 ± 0.309 | 3.223 ± 0.380 | 3.240 ± 0.314 | | 1.543 | 0.219 | |
| Insula | | Ins | | 7.504 ± 0.548 | 7.926 ± 0.737 | 7.852 ± 0.829 | | 0.352 | 0.704 | |
| Anterior Cingulate Cortex | | AntCingGy | | 4.110 ± 0.375 | 4.146 ± 0.553 | 4.193 ± 0.469 | | 0.332 | 0.718 | |
| Middle Frontal Gyrus | | MidFrontGy | | 19.605 ± 2.075 | 20.214 ± 2.361 | 20.342 ± 2.176 | | 0.243 | 0.784 | |
| Orbitofrontal Gyrus | | OrbFrontGy | | 11.824 ± 1.014 | 12.621 ± 1.226 | 12.422 ± 1.189 | | 0.984 | 0.3782 | |
| Inferior Frontal Gyrus | | InfFrontGy | | 9.131 ± 0.770 | 9.701 ± 0.998 | 9.462 ± 1.001 | | 0.482 | 0.619 | |
| Superior Frontal Gyrus | | SupFrontGy | | 29.367 ± 2.836 | 30.735 ± 2.993 | 30.770 ± 3.589 | | 0.386 | 0.680 | |

**Table S.1. Brain morphometry of the regions implicated in facial emotion recognition (FER) for each diagnostic group.** Gray matter volume (mean± standard devation, μl) calculated using voxel-based morphometric analysis is reported for each ROI drawn from the atlas labels of the n30r83 Hammersmith atlas. F and p-value are from an ANCOVA with age and total intracranial volume (TIV). P-values are false discovery rate-corrected for multiple comparisons. BD-I, bipolar disorder type I; BD-II, bipolar disorder type II; HC, healthy controls.

|  | | | | | | | | | | | | | | | | | | | | | | | | | | | |
| --- | --- | --- | --- | --- | --- | --- | --- | --- | --- | --- | --- | --- | --- | --- | --- | --- | --- | --- | --- | --- | --- | --- | --- | --- | --- | --- | --- |
|  | |  | | | | | | | |  | | | | | | | | | | | |  | | | | | |
|  | |  | | **BD-I** | |  | |  | | | | **BD-II** | |  | |  | | | **HC** | | | |  | | |  |  |
| **Node** | | **Betweenness** | | **Closeness** | | **Strength** | | **Betweenness** | | | | **Closeness** | | **Strength** | | **Betweenness** | | | **Closeness** | | | | **Strength** | | |  |  |
| Sadness |  | -0.899 |  | -2.233 |  | -2.343 |  |  | -0.294 | |  | | -0.867 |  | -0.784 |  |  | -1.033 | |  | -1.442 | | |  | -1.171 | |  |
| Neutral |  | -0.428 |  | -0.868 |  | -0.943 |  |  | -0.587 | |  | | -0.668 |  | -0.043 |  |  | -1.033 | |  | -1.900 | | |  | -1.276 | |  |
| Hippocampus |  | -0.899 |  | -0.448 |  | 0.071 |  |  | -1.174 | |  | | -0.909 |  | -0.052 |  |  | -0.323 | |  | 0.594 | | |  | -0.388 | |  |
| Amygdala |  | 1.455 |  | 0.012 |  | 0.738 |  |  | 0.000 | |  | | -0.129 |  | 1.438 |  |  | 2.044 | |  | 1.215 | | |  | 1.147 | |  |
| Fusiform gyrus |  | 1.141 |  | 0.814 |  | -0.313 |  |  | -0.881 | |  | | -1.072 |  | -1.458 |  |  | -1.033 | |  | -0.123 | | |  | -0.781 | |  |
| Insula |  | -0.742 |  | 0.812 |  | 0.733 |  |  | 0.000 | |  | | 0.749 |  | -0.509 |  |  | 1.334 | |  | 1.322 | | |  | 0.787 | |  |
| Anterior Cingulate cortex |  | -0.899 |  | 0.061 |  | 0.155 |  |  | -1.174 | |  | | -1.074 |  | -1.535 |  |  | -0.559 | |  | -0.668 | | |  | -1.150 | |  |
| Middle Frontal gyrus |  | 1.141 |  | 1.010 |  | 1.047 |  |  | 1.468 | |  | | 0.880 |  | 1.004 |  |  | 0.151 | |  | 0.193 | | |  | 1.423 | |  |
| Orbito-Frontal gyrus |  | -0.899 |  | 0.148 |  | -0.084 |  |  | 0.881 | |  | | 1.023 |  | 0.328 |  |  | -0.323 | |  | 0.210 | | |  | 0.096 | |  |
| Inferior Frontal gyrus |  | -0.114 |  | -0.507 |  | -0.190 |  |  | 0.000 | |  | | 0.280 |  | 0.522 |  |  | 0.387 | |  | 0.374 | | |  | 0.444 | |  |
| Superior Frontal gyrus |  | 1.141 |  | 1.199 |  | 1.128 |  |  | 1.762 | |  | | 1.787 |  | 1.088 |  |  | 0.387 | |  | 0.224 | | |  | 0.868 | |  |
|  | | | | | | | | | | | | | | | | | | | | | | | | | | | |

**Table S.2. Centrality measures per node for each diagnostic group.** Betweenness, Closeness, Strength indexes are reported. BD-I, bipolar disorder type I; BD-II, bipolar disorder type II; HC, healthy controls.
